# A Comprehensive Review: Biology of *Anopheles squamosus*, an Understudied Malaria Vector in Africa

**DOI:** 10.3390/insects16020110

**Published:** 2025-01-23

**Authors:** Valerie T. Nguyen, Dalia S. Dryden, Brooke A. Broder, Ayaan Tadimari, Primrose Tanachaiwiwat, Derrick K. Mathias, Panpim Thongsripong, Lawrence E. Reeves, Renee L. M. N. Ali, Mary E. Gebhardt, Kochelani Saili, Limonty Simubali, Edgar Simulundu, Douglas E. Norris, Yoosook Lee

**Affiliations:** 1 Florida Medical Entomology Laboratory, Department of Entomology and Nematology, Institute of Food and Agricultural Sciences, University of Florida, Vero Beach, FL 32962, USA; nguyen.v@ufl.edu (V.T.N.); daliadryden@ufl.edu (D.S.D.); ptanachaiwiwat@ufl.edu (P.T.); thongsripong.p@ufl.edu (P.T.); lereeves@ufl.edu (L.E.R.); 2 The W. Harry Feinstone Department of Molecular Microbiology and Immunology, The Johns Hopkins Malaria Research Institute, Johns Hopkins Bloomberg School of Public Health, Johns Hopkins University, Baltimore, MD 21205, USA; rali23@jh.edu (R.L.M.N.A.); douglas.norris@jhu.edu (D.E.N.); 3 Macha Research Trust, Macha 10101, Southern, Zambia; kochelani.saili@macharesearch.org (K.S.); edgar.simulundu@macharesearch.org (E.S.)

**Keywords:** Zambia, *Anopheles*, distribution, host association, vector, malaria

## Abstract

With ongoing global efforts to eliminate malaria, several countries have entered a pre-elimination stage, in which populations of the primary mosquito vector are reduced and the rates of malaria transmission are low. Efforts to eliminate malaria have changed the dynamics of disease transmission, causing a shift in anopheline community composition in some regions. One of the most abundant species of *Anopheles* found in pre-elimination areas, such as southern Africa, is *Anopheles squamosus* (Theobald, 1901; Diptera: Culicidae). Although *An. squamosus* has been documented across the African continent, limited information is available about its biology. Knowing what, when, and where the malaria vector bites humans can help develop effective malaria control strategies. The aim of this review is to compile the information available on *An. squamosus*’s biology, ecology, distribution, behavior, and potential role in disease transmission. The review of current literature suggested that *An. squamosus* is abundant throughout the African continent. Studies indicate that this species occupies the same range and exhibits similar behaviors to primary malaria vectors in southern and eastern African countries, like Zambia. In conclusion, *An. squamosus* continues to be an understudied species that has circumvented disease control measures and further studies are needed to develop effective control strategies.

## Simple Summary

*Anopheles squamosus* is a widespread mosquito species in Africa. Studies have shown that this species feeds on humans in indoor settings and is found to be infected with human malaria parasites. Despite this, *An. squamosus* continues to be an understudied species that has circumvented disease control measures. For this review, published literature, as well as online databases that reported *Anopheles squamous* occurrence in Africa were compiled. Our work indicates that *An. squamosus* is abundant throughout the African continent and exhibits similar behaviors to primary malaria vectors in southern and eastern African countries, like Zambia. Further studies are needed to develop resources to accurately identify this species from other mosquito species and to develop effective control strategies based on their biology and behavior.

## 1. Introduction

According to the World Health Organization (WHO), there were approximately 249 million malaria cases in the 85 countries where the disease is endemic and an estimated 608,000 deaths in 2022 [1]. The WHO has supported efforts to reduce the global burden of malaria by developing a framework for working toward elimination. The current malaria elimination strategies laid out in WHO guidelines for malaria (2024) include vector control, chemotherapies, and vaccines [2,3]. The current Global Technical Strategy for Malaria 2016–2030 (GTS) target is reducing malaria incidence and mortality rates by 90% and eliminating malaria in at least 35 countries by 2030 [4]. Between 2011 and 2021, there was an annual average reduction of malaria incidence of 25.4% [5]. However, this decrease was heterogeneous both within and across countries, and there was a 5% global increase in malaria incidence rates between 2019 and 2020, likely due in part to disruptions during the COVID-19 pandemic [2,6].

With global efforts to reduce the burden of malaria, several African regions, such as Cape Verde, central Senegal, Guinea-Bissau, Isle of Príncipe, and southern Zambia, reached the pre-elimination stages of malaria transmission [7,8,9,10,11,12,13]. The pre-elimination status is defined by a population with either a rapid diagnostic test (RDT) positivity rate below 5% annually or a parasite positivity rate lower than 5% among those with a fever [14]. The decrease in global malaria cases has been largely attributed in part to vector control that utilizes tools, such as insecticide-treated bed nets (ITN) and indoor residual spraying (IRS), which target indoor-foraging (endophagic) and indoor-resting (endophilic) mosquitoes [15,16]. As a result of the implementation of these control methods, many of the principal vector species of malaria, such as *Anopheles gambiae* sensu lato (s.l.), have been declining in abundance in some countries, like Kenya, Tanzania, and Zambia [17,18,19,20].

Identification of the knowledge gaps must be done to adequately assess vector-borne pathogens across the African continent. There is a need for improved infrastructure, an understanding of the ecology of vector species and the environmental conditions that impact them, to assess their role in disease transmission [21,22,23]. For example, in Zambia, the need for vector ecology studies could be alleviated by improving the understanding of the human–vector contact frequency, which is pivotal for assessing disease transmission in vector-borne systems and planning mitigation measures [21]. To adequately assess trends in vector-borne diseases in Africa, there must be an effort to increase the capacity for research on vector-borne zoonotic diseases by enhancing the interdisciplinary focus of research and building stronger communications between countries [24].

Despite these achievements and the identification of areas for improvement, the final elimination of malaria from many regions and countries (e.g., southern Zambia, northeastern South Africa, Botswana, and Namibia) is now proving to be frustratingly difficult, and such locations remain static in the pre-elimination stage. Tenacious residual malaria infections persist in these pre-elimination zones despite access to rapid diagnostics and treatment, comprehensive IRS campaigns, and the widespread distribution of ITNs [14]. Despite the prolonged extirpation or decline of major vector species, persistent residual malaria has prompted many involved in malaria control to suspect that understudied secondary vector species contribute to the remaining malaria transmission in pre-elimination zones [25,26]. These residual malaria cases highlight that existing vector control tools that have focused on the major vector species alone are not sufficient to eliminate malaria from some regions [27].

Species often considered secondary vectors have been implicated in malaria transmission in pre-elimination zones and are often understudied. Examples include *An. vaneedeni* and *An. parensis* (members of the *An. funestus* group) in southern Africa and *An. coustani* and *An. ziemanni* in central Africa [28,29,30,31]. An expanding body of work has implicated *An. squamosus*, *An. rufipes*, and *An. coustani* in malaria transmission in Zambia, especially in pre-elimination settings [17,26,32,33,34]. *Anopheles* collections in Zambia revealed morphological misidentification and underrepresentation of many anopheline species in sequence databases, which confounds efforts to confirm the identity of potential malaria vector species [35]. Therefore, continued development of methodologies that allow more accurate species identification of these understudied malaria vector populations is critical for understanding malaria transmission in the pre-elimination setting and elucidating the potential role of the understudied secondary vectors in residual malaria cases.

Studies of malaria transmission dynamics in pre-elimination settings are particularly relevant to discussions of gene drive and other novel vector control strategies. For example, the ITN, one of the most successful malaria control tools, was devised with the understanding that the primary malaria vector bites humans at night indoors while people are sleeping [36]. Other *Anopheles* species that bite humans outdoors could circumvent control measures, like ITNs. Other regions in Africa will inevitably reach the malaria pre-elimination phase at some future time through the implementation of available and perhaps new control strategies. Understanding the behavior and ecology of understudied malaria vectors will be critical in advancing comprehensive malaria control strategies targeting multiple vector species that can lead to malaria elimination.

*Anopheles squamosus* is a secondary vector species of malaria that can be found across sub-Saharan Africa [37]. This species is currently understudied because it is believed to forage outdoors (exophagic) and to be primarily associated with non-human animal hosts (zoophilic) [26]. These are based on limited studies and have created a perception that it does not pose a threat to public health. However, studies have shown that *An. squamosus* will opportunistically feed on humans and can be present around human dwellings [23,26]. Moreover, this species has been found naturally infected with *Plasmodium falciparum*, a causative agent of human malaria, and is abundantly collected during mosquito surveys [32,38].

Another barrier to studying *An. squamosus* is the morphological identification and separation of this species from other difficult to distinguish African anophelines. Many other anophelines, like *An. gambiae* s.l., exist as complexes of morphologically similar species with members exhibiting variable vector capability [32,39], insecticide resistance [40], and host association [41,42]. Therefore, the ability to distinguish exact species within an anopheline group is critical for assessing the associated malaria risk and planning effective interventions targeted to the particular species of concern. As adults, *An. squamosus* is morphologically identical to *An. cydippis* [43], and there are at least five chromosomal forms based on chromosome inversions that may correspond to cryptic species under the name *An. squamosus* [44]. Unless genetic methods are used to distinguish these taxa, any study of these species is based on an uncertain amalgamation of these cryptic species.

Creating further challenges to studying this and other understudied malaria vectors is the diversity and morphological similarity among Afrotropical anophelines [44]. Distinguishing African anophelines from one another often requires specimens with morphological characters intact, which can be challenging because of the methods and logistics of field collections [32,39,40,41,42].

Existing knowledge of *An. squamosus* is scattered across multiple articles published over decades, some of which are not digitized or available online, which poses a challenge to accessing and compiling what is known about this species. As of September 2024, only 53 results are found when searching “*Anopheles squamosus*” in PubMed. The objective of this review is to present a summary of the literature recovered on the identification, distribution, ecology, and biology of this species. In addition, it aims to shed light on the knowledge gaps regarding *An. squamosus* as an understudied malaria vector and to inspire future research on this and other understudied *Anopheles* species in Africa.

## 2. Materials and Methods

**Figure 1:**
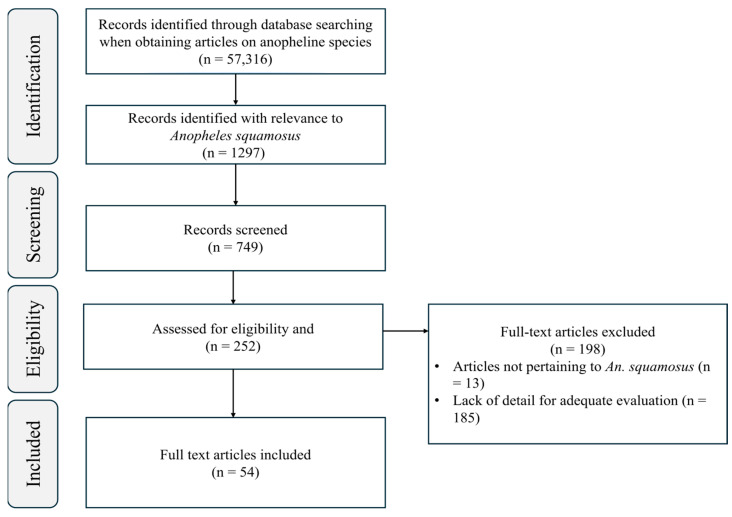
PRISMA [45] flow diagram of the literature review process for studies of *An. squamosus*.

We conducted literature searches for *An. squamosus* using online search engines including PubMed, Science Direct, Google Scholar, and the National Center for Biotechnology Information (NCBI) nucleotide database. Term searches ranged from general terms, such as “*Anopheles*”, to specific terms such as “*Anopheles squamosus*”. In each search engine, the following search terms were used: “*Anopheles*”, “*Anopheles squamosus*”, “*An. squamosus*”, “*A. squamosus*”, “*Anopheles squamosus* AND *Plasmodium falciparum*”. We provided a flow diagram of our literature review process following the PRISMA 2020 guidelines in Figure 1 [45]. We investigated the literature and extracted all available records of *An. squamosus* and other *Anopheles* spp. found together with *An. squamosus* in an Excel spreadsheet (see Supplementary Material Table S1). Additional species occurrence records were also retrieved from the Africa Vector Database [46]. For categorizing each country to subregions in a continent, we used the geographic regional groups according to UN M49 Standard country and area codes for statistical use [47]. For example, UN M49 Standard classifies Africa into 4 subgroups, namely Eastern, Middle, Southern, and Western Africa.

The distribution map of *An. squamosus* was created using QGIS software version 3.30.2 [48]. The basemap of African continent topography in the public domain was obtained from Natural Earth Tiles [49].

Representative images of *An. squamosus* morphology in Zambia were created using adult female specimens collected along the outer perimeter of a goat pen using mouth aspirations at the Hanatanga Village in the Ngolwe zone of southern Zambia (16°12′26.766″ S; 27°0′1.554″ E) in January 2024. This site was selected because *An. squamosus* has been collected in high abundance by the Macha Research Trust team in their previous malaria vector surveillance studies [32,33]. Collected *An. squamous* were frozen at 0 °C overnight and mounted to a glass microscope slide within 24 h. A total of three *An. squamosus* samples in the best conditions were used for photography. The mounted mosquito was photographed with a Canon 5D SR digital SLR camera using a focus-stacking system that consisted of a 5× or 10× Mitutoyo infinity-corrected microscope objective attached to a Canon 200 mm L prime lens. The camera and lens were mounted onto an automated StackShot rail (Cognisys Inc., Traverse City, MI, USA). The camera was moved so that the mosquito specimen was just out of focus, and then moved in increments of either 7 µm (5×) or 15 µm (10×), with one photograph taken between each step, until the mosquito was out of focus in the opposite direction. The raw images were stacked (i.e., the in-focus areas of each image were digitally merged to create one image with the entire specimen in focus) using Helicon Focus (v 8.2.13) program and then edited to clean the background using Adobe Photoshop (v 25.5.1).

## 3. Results

### 3.1. Anopheles squamosus Identification

**Figure 2:**
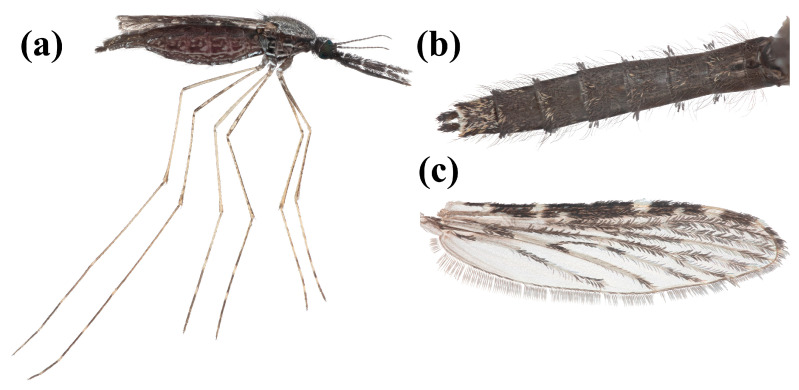
Stacked focus image of *An. squamosus* taken by Dr. Lawrence E. Reeves and processed by Valerie T. Nguyen. (**a**) Lateral view of a blood-fed, (**b**) abdomen tufts, and (**c**) wing of *An. squamosus* female.

In 1901, Theobald described *An. squamosus* as “a very pronounced scaly species, not like any other *Anopheles* I have ever seen” [37]. As an adult, *An. squamosus* can be identified by several key features from other *Anopheles*, except for *An. cydippis*, as described in several morphological identification keys [44,50,51]. The first distinguishing feature is the presence of laterally projecting tufts of abdominal scales (Figure 2b). *Anopheles squamosus* is described by Evans [50] as being predominantly black with contrasting pure white scales. Females have shaggy palpi with four narrow bands that are white, black, or bronzy-brown dorsal scales, and their last dorsal segment has numerous white scales (Figure 2a).

A study to assess the accuracy of identifying anopheline species found that only 37% of adult *An. squamosus* were identified correctly by morphology [30]. Distinguishing this species must be done in their 4th instar larval stage or through molecular tools. However, utilizing larval keys to differentiate *An. squamosus* from *An. cydippis* is challenging.

Currently, *An. squamosus* makes up only 0.6% of the literature on anopheline species when searching PubMed, Science Direct, and Google Scholar. In addition, there is a major lack of genomic resources for this species within NCBI, with only 210 genetic sequences as of September 2024. Furthermore, no cytochrome C oxidase subunit I (COI) sequences of *An. cydippis* are available, and the internal transcribed spacer 2 (ITS2) assay used by many researchers to distinguish cryptic anopheline species does not reliably amplify *An. squamosus*/*An. cydippis* DNA [38].

### 3.2. Anopheles squamosus Distribution

**Figure 3:**
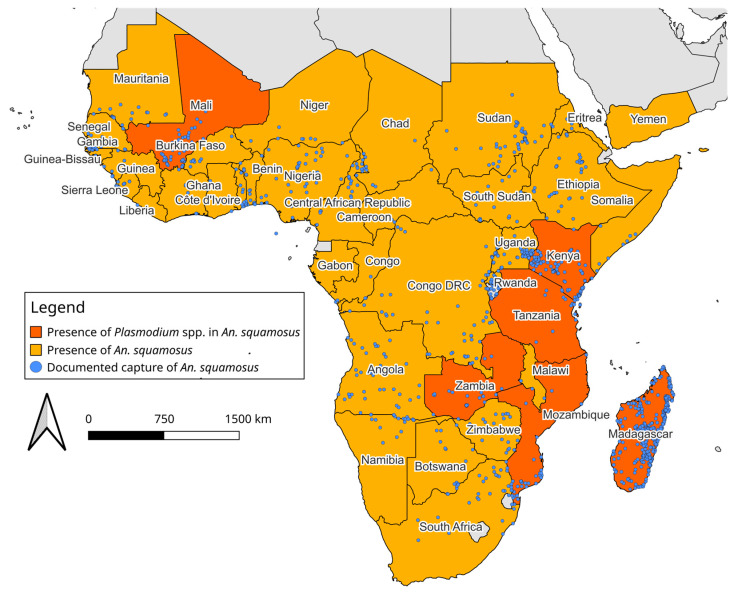
*Anopheles squamosus* distribution across Africa and West Asia. Highlighted countries are documented presence of *An. squamosus* collected from WRBU and Kyalo et al. [46] and countries where *Plasmodium* spp. has been identified in this species. The points on the map indicate coordinate data where *An. squamosus* has been captured.

In 1901 The current literature describing the distribution of these species varies throughout historical documentation. Theobald [37] describes this species as being native to the Middle African region in 1907. Evans [50] states that the distribution of *An. squamosus* is broad, covering Western, Eastern, and Southern Africa in 1927. Similarly, De Meillon [52] describes its distribution as widespread and practically across the whole continent in 1951. The literature review from this study suggested that *An. squamosus* is widespread across the African continent, consistent with the findings of De Meillon [52]. This species has been documented in 41 African countries across entomological surveys since 1898, as described in the Africa Vector Database, Updated list of *Anopheles* species (Diptera: Culicidae) by country in the Afrotropical Region and associated islands by Irish et al. [53], and by other anopheline distribution descriptions (Table 1 and Table S1). The Africa Vector Database contains an inventory of anopheline species in Africa between 1898 and 2016 created by Kenyan Medical Research Institute and Wellcome Trust collaborators to document anopheline species occurrence. The data compiled from this database in addition to literature published after 2016 identified 1331 unique geographic coordinate points where *An. squamosus* presence has been reported [17,23,32,33,34,46,54,55,56,57,58,59,60,61,62,63] (Figure 3).

The first documented capture of this species occurred in 1898 in Sierra Leone (Western Africa). Two years later, it was documented in eastern Africa, 5765 km away. By 1904, *An. squamosus* was documented in Africa’s Eastern, Middle, Southern, and Western regions. Identifying this species at distance points over a short period indicates that this species was widespread throughout Africa before 1898. The known distribution of *An. squamosus* based on these data reaches its northern limit at Adrar, Mauritania (20.511° N, −13.049° E) [46] and extends as far south as Northern Cape, South Africa (−30.452° S, 21.228° E) [39]. Its most eastern occurrence has been documented in the Sava region of Madagascar (−15.2428° S, 50.4434° W) [46] and the furthest western report was from Thies, Senegal (14.801° N, −16.926° E) [46].

Kuznetsov [64] described the only detection of *An. squamosus* outside of the African continent in the country of Yemen. This observation occurred in 1965 during larval sampling in the coastal plain of Tihama in quick-drying pools that are not ideal for the complete lifecycle of anopheline species. A total of 380 larvae were documented in this survey, with one larva being identified as *An. squamosus*. Since this documentation, there has not been another published occurrence of *An. squamosus* outside of Africa. It remains to be determined if this species is reproductively established in Yemen and other western Asian countries.

**Figure 4:**
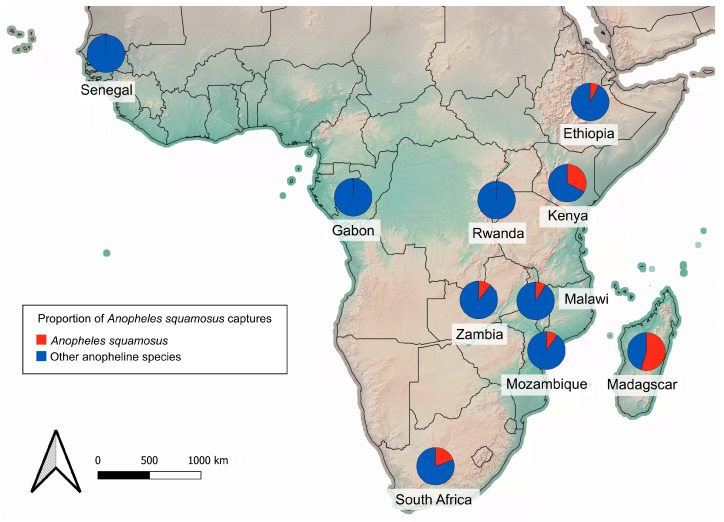
Proportion of *An. squamosus* captured across 25 anopheline trapping studies conducted between 1980 and 2022. The basemap of African continent topography is obtained from Natural Earth Tiles [49].

*Anopheles squamosus* can be found in abundance throughout much of the known distribution. Anopheline surveillance was informative at providing a snapshot of the community composition of potential vectors inhabiting a given location. Across 25 studies in 9 countries (Ethiopia, Kenya, Madagascar, Malawi, Mozambique, Rwanda, Senegal, South Africa, and Zambia) primarily in eastern and southern Africa, *An. squamosus* comprised as low as 0.1% and as high as 68.0% of adult anopheline trap captures (Figure 4, Table 2) [23,26,29,30,33,38,39,41,42,54,56,57,59,60,65,66,67,68,69,70,71,72,73,74]. These studies consisted of both indoor and outdoor surveys using various methods of collection (human-baited traps, animal-baited traps, CDC light traps).

The widespread distribution of *An. squamosus* is similar to that of the primary malaria vectors in Africa, such as those in the *An. gambiae* and *An. funestus* groups [46]. Based on the data in the African Vectors Database, *An. squamosus* was captured most often alongside species in the *An. gambiae* complex, *An. coustani*, and *An. funestus* complex [46]. The occurrence of *An. squamosus* in regions where other vectors actively transmit malaria suggests that this vector has potential exposure to circulating parasites and may sustain malaria transmission when primary vectors are reduced or eliminated.

### 3.3. Larval Biology of Anopheles squamosus

Identification of larval habitats for *An. squamosus* is essential, as they can be morphologically distinguished from *An. cydippis* in this life stage [38]. In addition, control of malaria vectors in the larval stage can be more effective in certain settings due to their inability to escape the habitats [62,78,79]. Since indoor targeted malaria control for adult anophelines is ineffective against exophagic vectors, targeting these species’ larval stages could be an effective alternative control strategy. However, little larval biology is known for this species. One study was conducted to obtain F1 progeny from wild populations of *An. squamosus* in Madagascar and 35.6% of wild females successfully oviposited in tubes or cages [80]. However, this study did not record the successful hatch rate from these eggs to larvae. No other documented colonization attempt of this species is available to date. Consequently, the duration of larval or adult life span is unknown for this species.

*Anopheles squamosus* larvae have been recorded in naturally occurring (ponds, rivers, and lagoons) and human-created (rice fields, irrigation drains, and tire tracks) bodies of water [73]. They are often associated with six other African anopheline species in these larval habitats (*An. gambiae* s.l., *An. funestus* s.l., *An. coustani*, *An. cinereus*, *An. demeilloni*, and *An. pharoensis*) [56,81]. In swamps of Ethiopia, they have been found to coexist in high numbers with *An. pharoensis* [67]. The highest contributing factors for the presence of *An. squamosus* larvae are high vegetation and algae [67]. There is also a positive correlation of *An. squamosus* with shallow depths of water [56]. In Ethiopia, Kenea et al. [67] found that *An. squamosus* larvae were located further away from human dwellings. In contrast, Adugna et al. [56] reported a positive correlation of *An. squamosus* larval habitats being closer to human dwellings. The studies on larval habitats and species associations were spatiotemporally sparse and only completed in eastern Africa. Therefore, the reported findings and any conclusions drawn from them are extremely limited.

There is very little known on the microbial tolerance of *An. squamosus*. *Anopheles squamosus* larvae have been observed to be more tolerant than the *An. gambiae* complex when exposed to fungal biocontrol agents, such as *Coelomomyces* [81]. *Coelomomyces* fungus can infect mosquito larvae and prevent pupation while other organisms remain unaffected [82]. Muspratt found that *Coelomomyces* fungus was highly effective for controlling *An. gambiae* larvae (>95% mortality) [81]. However, when *An. squamosus* was exposed to the same *Coelomomyces* fungus, there was a very low percentage (8% infection rate) of larvae infected with the biocontrol agent [81]. This suggests that *An. squamosus* may behave differently from principal malaria vector species when exposed to such biological control agents, and studies of immune response based on the well-studied principal malaria vectors may not translate to other anopheline species. There are no other studies testing additional microbial larvicides on *An. squamosus* reported as of December 2024.

### 3.4. Adult Anopheles squamosus Behavior

Understanding vector behavior is critical for disease control, as these vector behaviors can be interrupted to effectively reduce pathogen transmission [83,84]. Approximately 30–40 of the 430 described *Anopheles* species are currently known to be vectors of human pathogens [85]. A key behavioral trait that primary human malaria vectors exhibit is the propensity to feed on people indoors at night [83,86]. Targeting this behavior using ITN and IRS campaigns has drastically reduced malaria cases in many areas [29,36]. However, this has caused changes in the behaviors of primary vectors in some countries and shifts in the anopheline community composition in others [17,20,87,88].

Knowledge on the behavior of adult *An. squamosus* is scarce and can be attributed to the lack of research focused on this species. This species is commonly associated with livestock and considered zoophilic, which has made it less of a concern for human health initiatives [26]. In the low transmission areas of Mozambique, *An. squamosus* predominantly exhibits exophagic behavior [29], and they were reported to forage in the early evening in one study in Zambia [26].

Despite the notion that *An. squamosus* adults express exophagic behavior, this species has been recorded in indoor settings. In high transmission areas in Mozambique, up to 68.4% of *An. squamosus* were captured host-seeking indoors, predominantly in the evening before human occupants went to bed and while they were in bed [29]. The close proximity of *An. squamosus* to humans can increase instances of malaria transmission due to their potential ability to vector human *Plasmodium* parasites [89]. This species has been captured using CDC light traps, human landing catches (HLC), human-baited traps, and livestock-baited traps. Seven trapping studies were identified during our literature review that collected *An. squamous* or *An. cydippis* indoors. Across these studies, up to 41% of trap captures consisted of species identified as *An. squamosus* [29,33,38,55,56,65,66] with an average of 14.7% of indoor trap captures identified as *An. squamosus*.

Historically, this species has been considered zoophilic [37], as *Anopheles squamosus* has demonstrated a preference for feeding on non-human hosts if they are present [26]. However, there is evidence that feeding behavior for *An. squamosus* varies depending on its location. Across several studies, there were observed differences in HLC in different countries, which may be attributed to the availability of alternate hosts [23,30,55,66,74]. The recorded host species for *An. squamosus* include sheep, cow, pigeon, chicken, goat, dog, pig, and human [26,32,33,39,56,57]. From five host association studies between 2011 and 2022, where 670 specimens were collected, the host most often found for *An. squamosus* was cow (n = 250), followed by goat (n = 158), non-human (n = 65), pig (n = 60), cow and goat (50), cow and human (n = 40), human (n = 26), cow and pig (n = 12), dog (n = 5), human and animal (n = 3), and chicken (n = 1) (Table 3) [26,32,33,56,57]. From these studies, a total of 10.3% of the blood meals contained human blood, indicating that this species readily feeds on humans across habitats. In Macha, Zambia, *An. squamosus* had a higher rate of reported anthropophily when compared to Cameroon, Kenya, and Senegal based on indoor CDC light trap collections [26].

### 3.5. Anopheles squamosus Contribution to Pathogen Transmission

*Anopheles squamosus* has been implicated as a potential pathogen vector in Zambia, Madagascar, Mozambique, Namibia, Mali, Kenya, and Tanzania (Table 4) [26,29,46,68,69,73,90,91]. Although it is considered a secondary vector species of certain human diseases, such as malaria, secondary vector species may become primary vectors under certain conditions [92]. Distinguishing features of a primary malaria vector include their relative abundance, a high propensity to feed on humans, and sporozoite rate [93]. Most of the published studies on *An. squamosus* is focused on Zambia and Madagascar. Despite being identified across sub-Saharan Africa, pathogen transmission is unique to a location, as environmental and socio-economic factors impact transmission, which can vary from country to country [94].

*Anopheles squamosus* has been found infected with human *Plasmodium* sporozoites in Kenya, Mali, Mozambique, Madagascar, Tanzania, and Zambia [29,32,33,46,88]. Reported infection rates are variable due to sample size; however, these reports verify that *An. squamosus* feeds on humans frequently enough to be infected with infectious human-only parasites. The first detection of *Plasmodium* sporozoites in *An. squamosus* was in Tanzania in 1964, where a single mosquito was infected with the parasite [96]. In a study in Mozambique, *An. squamosus* had the highest *Plasmodium* sporozoite infection rate of 5.8%, more than *An. parensis*, *An. arabiensis*, and *An. funestus* [29]. In a trapping study in Zambia, all three captured *An. squamosus* samples were found to be infected with *P. falciparum* [33]. In Madagascar, two distinct strains of *P. vivax*, another causative agent of human malaria, were identified in *An. squamosus* [57].

Rift Valley fever virus (RVFV) is a virus that causes a mosquito-borne viral zoonosis affecting humans and livestock. RVFV can cause severe disease in animals, while severe symptoms in humans are rare [99,100]. *Aedes* mosquitoes are recognized as the primary vector for RVFV; however, *Anopheles* and *Culex* mosquitoes are considered secondary vectors [99]. Some anopheline species can maintain RVFV and transmit it to their offspring [68], and in Madagascar, RVFV was detected in five pools of *An. squamosus*, suggesting this species could be a host of RVFV [68].

Bluetongue disease, caused by bluetongue virus (BTV), is transmitted predominantly by *Culicoides* biting midges among ruminant animals. It causes a wide array of symptoms for ruminant animals but distinctly causes severe swelling of the tongue [101]. A survey of mosquitoes in Madagascar identified a pool of *An. squamosus* that tested positive for BTV [98]. Although it does not indicate that this species is a competent vector of BTV, it demonstrates that these mosquitoes can acquire the virus from infected animals. Further research will be needed to determine any roles for *An. squamosus* in maintenance and transmission of BTV.

### 3.6. Genetic Information of Anopheles squamosus

As mentioned in the *An. squamosus* identification section, there is limited DNA sequence data available for *An. squamosus* [102]. There is no DNA sequence available for *An. cydippis*, a species that shares identical adult morphology. Therefore, the capacity to develop *An. squamosus*-specific species identification assay is limited.

There are ongoing efforts to overcome these barriers, including the authors of this paper [102]. The first complete mitogenome sequence (Genbank Accession # OP776919) and nuclear genome sequence data (NCBI Sequencing Reads Archive accession number WRR22114392) became available in 2023. The *An. squamosus* mitogenome sequence was closely matched to the *An. gambiae* mitogenome sequence (Genbank Accession # L20934) with 91.5% sequence similarity. The high-throughput short reads sequencing of *An. squamosus* allowed the assembly of a contig containing the intergenic transcribed spacer 2 (ITS2) region [102], and the contig sequence is available on Genbank (Accession # OQ241725).

## 4. Discussion

*Anopheles squamosus* has been documented in 41 of the 54 currently recognized African countries, with many studies reporting a robust number of this species [103]. These collection records indicate that this species was likely widespread and well-established long before their first documentation in the early 1900s. The broad distribution of *An. squamosus* is similar to other primary malaria vectors in Africa, such as *An. gambiae*, *An. arabiensis*, and *An. funestus*, suggesting that *An. squamosus* has a reasonable chance of coming into contact with human hosts and infectious parasites. In Zambia, this species exhibited a high human blood index, even in the presence of livestock [26]. In addition, they have been found to be infected with human *Plasmodium* sporozoites [34,97]. These combinations of factors suggest that *An. squamosus* may play a more significant role in malaria transmission than previously recognized.

*Anopheles squamosus* poses a challenge for malaria transmission [25,96,104]. due to its exophilic nature, which allows them to evade indoor-based malaria intervention methods such as ITN and IRS. As a result, humans are more likely to encounter *An. squamosus* and other secondary vector species in outdoor settings when outdoor-based interventions are not put in place [36,87]. For example, in Mozambique, there was an increase in outdoor *An. squamosus* population numbers [29] after an increase in IRS campaigns. This suggests a potential behavioral adaptation or shift in mosquito community populations in response to indoor malaria control. Therefore, targeted control of primary endophagic vectors through ITN and IRS has the potential to shift vector status in favor of exophagic vectors, like *An. squamosus* [105]. This highlights the need for comprehensive control approaches that address both indoor and outdoor transmission risks.

The public health significance of *An. squamosus* cannot be understated, as it has also been identified as a potential vector of RVFV, and BTV due to zoophilic behavior. For *An. squamosus*, records indicate a high vector abundance, a propensity to feed on humans, and positive *Plasmodium* sporozoite rates, thus indicating that this species is a competent vector for malaria. Tackling the primary malaria vector remains the highest priority for many malaria-endemic countries. However, the lack of knowledge on understudied malaria vectors places major barriers in developing effective malaria elimination strategies.

Despite its significant role in malaria transmission, there is a lack of reliable molecular identification tools, such as internal transcribed spacer (ITS) polymerase chain reaction (PCR), for *An. squamosus*. The current molecular identification of *An. squamosus* based on Sanger sequencing of Cytochrome Oxidase I is not ideal for routine surveillance. Since *An. squamosus* is morphologically identical to *An. cydippis* in their adult life stages, distinguishing between these cryptic species becomes challenging without robust molecular species identification tools [106]. This represents the key barrier to gaining further understanding of the biology and behavior of this species. Therefore, there is a pressing research need for building more genomic resources to improve our understanding of and enhance surveillance for *An. squamosus*. Some efforts are underway to address this research need by assembling the mitogenome and nuclear genome of *An. squamosus* [102]. Further studies comparing the *An. squamosus* ITS2 sequences with those from other *Anopheles* species may facilitate the development of a low-cost molecular species identification assay suitable for routine malaria vector surveillance. Sequencing data for *An. cydippis* may also facilitate the identification of the two species at adult stages, allowing more in-depth analysis on the similarity and differences on the bionomics of the two species.

Past studies that identify *An. squamosus* on morphology alone may not be accurate [35]. In our effort to create a reliable distribution map, we attempted to separate records verified only by morphological examination from records confirmed by both morphological and molecular methods. However, the lack of detailed metadata for many of the records and publications prevented accurate categorization. This further emphasizes the need for cost-effective tools that will help enhance species identification, allowing further investigations to inform on the role of *An. squamosus* in pathogen transmission and development of disease mitigation strategies.

## 5. Conclusions

*Anopheles squamosus* is a widespread mosquito species found abundantly across the African continent (41 of the 54 African countries). Despite their implication in malaria transmission and potentially vectoring other diseases impacting human and animal health, research and information on this species remains scarce. Historically, *An. squamosus* has been regarded as an exophagic and zoophilic species, and as a result, they are not viewed as a threat to public health. However, our review indicates that this species occupies the same range and exhibits similar behaviors to primary malaria vectors, as they have been implicated as feeding on humans in indoor settings and found to be infected with human *Plasmodium* parasites. The larval biology of this species is largely unknown, and genetic information for this species is very limited. There is no reliable molecular species identification assay that can positively identify *An. squamosus* to date. Further research to gain more information on their biology, behavior, and genetics is needed for developing appropriate vector control strategies. Ultimately, such new strategies can be added to the existing malaria elimination framework for countries and regions in pre-elimination stages.

## Figures and Tables

**Table 1:** Geographic region, country and year of first documented occurrence of *An. squamosus.* The geographic regions are according to UN M49 Standard country and area codes for statistical use [47].

Geographic Region	Country	First Documented Occurrence
Eastern Africa	Eritrea	1941
	Ethiopia	1920
	Burundi	1935
	Kenya	1900
	Madagascar	1902
	Malawi	2012
	Mozambique	1901
	Rwanda	1933
	Somalia	1951
	South Sudan	1903
	Tanzania	1902
	Uganda	1907
	Zambia	1941
	Zimbabwe	1901
Middle Africa	Angola	1904
	Cameroon	1907
	Central African Republic	1950
	Chad	1950
	Republic of Congo	1943
	Democratic Republic of the Congo	1902
	Gabon	1999
Northern Africa	Sudan	1903
Southern Africa	Botswana	1961
	Eswatini	1974
	Namibia	1950
	South Africa	1905
Western Africa	Benin	1950
	Burkina Faso	1948
	Cote d’Ivoire	1950
	Gambia	1902
	Ghana	1911
	Guinea	1950
	Guinea-Bissau	1946
	Liberia	1902
	Mali	1909
	Mauritania	1945
	Niger	1961
	Nigeria	1909
	Senegal	1908
	Sierra Leone	1898
	Togo	1902
Western Asia	Yemen	1965

**Table 2:** Mean percent of *An. squamosus* collected in anopheline trapping studies grouped by country. Geographic regions are according to UN M49 Standard country and area codes for statistical use [47].

Geographic Region	Country	Mean % *An. squamosus*	% *An. squamosus* Range	References
Eastern Africa	Ethiopia	7.0	0.34–27.2	[23,56,59,67]
	Kenya	38.8	0.26–68.0	[41,72,75]
	Madagascar	68.0	2.76–97.4	[57,66,68,71,73,76]
	Malawi	9.5	-	[60]
	Mozambique	8.7	2.3–32	[29]
	Rwanda	0.004		[74]
	Zambia	10.0	0.5–41.4	[45]
Middle Africa	Gabon	0.23	-	[77]
Southern Africa	South Africa	18.8	0.12–100	[39,54,70]
Western Africa	Senegal	0.1	-	[42]

**Table 3:** Identified bloodmeals across five studies between 2011 and 2022.

Identified Blood Meal	Sample Size	Proportion (%)	References
Chicken	1	0.1	[57]
Human and Animal	3	0.5	[33]
Dog	5	0.8	[33]
Cow and Pig	12	1.8	[57]
Human	26	3.9	[33,57]
Cow and Human	40	6.0	[56,57]
Cow and Goat	50	7.5	[33]
Pig	60	9.0	[26,33]
Non-human	65	9.7	[33]
Goat	158	23.6	[26,33,38]
Cow	250	37.3	[26,33,38,56]
Total	670		

**Table 4:** Diseases to which *An. squamosus* has been implicated as a potential vector.

Disease/Pathogen	Detection Method	Country	References
Malaria	ELISA, Salivary gland dissection	Kenya, Madagascar, Mali, Mozambique, Namibia, Tanzania, Zambia	[29,33,46,95,96,97]
Rift Valley fever	PCR	Madagascar	[68]
Bluetongue	Indirect Immunofluorescence assay	Madagascar	[98]

## Data Availability

No new data were created in this study.

## Supplementary Materials

The following supporting information can be downloaded at: https://www.mdpi.com/article/10.3390/insects16020110/s1, Table S1: Records of *Anopheles squamosus* occurrences.
